# Relationship Between Menstrual Phase-Induced Sleep Variations and Performance in Elite Female Rowers

**DOI:** 10.7759/cureus.109839

**Published:** 2026-05-28

**Authors:** Prashu Ethirajan, Sai Vineet Damodar Premkumar, Sai Aditya Raman, Rohit K R, Thiagrajan K A, Arumugam Sivaraman, Vignesh Ram Kasthuri, Nithila Sundresh

**Affiliations:** 1 Department of Arthroscopy and Sports Medicine, Sri Ramachandra Institute of Higher Education and Research, Chennai, IND

**Keywords:** female athletes, menstrual cycle, performance variability, rowing, sleep efficiency, wearable monitoring

## Abstract

Background

Hormonal variations inherent to the menstrual cycle have been shown to affect recovery physiology in female athletes; however, their integrated effects on sleep architecture and sports performance remain poorly elucidated. Emerging evidence suggests that sleep quality varies across menstrual phases, with low-hormone states predisposing to sleep disturbance and impaired recovery. Given the role of sleep in neuromuscular function, recovery, and high-intensity performance, such variations may cause performance decrements, especially in physiologically demanding sports such as rowing. Despite this, current athlete monitoring frameworks do not evaluate menstrual cycle status and sleep parameters together. There are also limited studies in this regard among Indian female rowers. This study aims to evaluate menstrual phase-specific variations in sleep efficiency and rowing performance in elite female rowers.

Methods

This prospective observational study was conducted at a private sports medicine facility in South India. After obtaining informed consent, 15 elite female rowers aged between 18 and 25 years were monitored across three menstrual phases for nine consecutive menstrual cycles. Menstrual-cycle phases were categorized as high-estrogen late follicular, high-progesterone early luteal, and low-hormone early follicular plus late luteal phases. Sleep efficiency (%) was calculated from sleep metrics (duration of rapid eye movement (REM) sleep, awake periods, light sleep, deep sleep) recorded from Garmin (Garmin Ltd., Olathe, KS) wearable devices. Performance was measured by a standardized 500-m ergometer time trial. Phase-wise differences were assessed using Friedman repeated-measures analysis, with chi-square (χ²) reported as the test statistic and Kendall’s W as the effect size. Pairwise within-athlete comparisons between high-estrogen and low-hormone phases were performed using the Wilcoxon signed-rank test. Pearson correlation assessed the association between sleep efficiency and 500-m performance time using pooled phase-level observations.

Results

Mean sleep efficiency was significantly (p < 0.001) higher in the high-estrogen phase (96.90 ± 1.90%), followed by the high-progesterone phase (94.20 ± 2.30%) and lowest in the low-hormone phase (88.70 ± 2.80%). Performance was significantly (p < 0.001) reduced in the low hormone phase (119.80 ± 3.00 s), followed by the high progesterone phase (115.80 ± 2.40 s), with the best performance in the high estrogen phase (111.20 ± 2.80 s). Sleep efficiency showed a strong negative correlation with performance time (r = -0.86, p < 0.001), indicating that low sleep efficiency affects performance.

Conclusion

Menstrual cycle phase-specific variations significantly influence sleep efficiency and rowing performance, with low-hormone phases demonstrating the most unfavorable sleep-performance profile. These findings support the integration of menstrual cycle tracking with objective sleep monitoring to inform phase-specific, individualized training and recovery strategies for optimizing performance in elite female athletes.

## Introduction

Female athlete physiology remains underrepresented in sports medicine research, with a continued reliance on male-derived evidence to guide training, recovery, and performance strategies [1,2]. This limitation is particularly relevant given the dynamic endocrine milieu in female athletes, where cyclical fluctuations in estrogen and progesterone exert systemic effects on neuromuscular function, metabolism, thermoregulation, and sleep regulation [3].

The menstrual cycle is increasingly recognized as a key biological determinant of athletic performance; however, existing evidence remains heterogeneous and mechanistically incomplete. High-estrogen phases, particularly the late follicular phase, have been associated with enhanced neuromuscular efficiency and improved exercise tolerance, whereas low-hormone phases may predispose athletes to increased fatigue and reduced performance capacity [3,4]. Despite these observations, the pathways linking hormonal variation to performance outcomes remain incompletely understood.

Sleep is a fundamental pillar of athletic recovery and performance optimization. Normal sleep architecture comprises non-rapid eye movement (NREM) sleep, including slow-wave sleep, and rapid eye movement (REM) sleep, which cycle across the night in a highly regulated manner [5]. Slow-wave sleep is integral to physical restoration through growth hormone secretion and metabolic recovery, whereas REM sleep supports neurocognitive processes such as motor learning and memory consolidation [6]. In elite athletes, even subtle disruptions in sleep architecture - manifested as reduced REM sleep, impaired sleep efficiency, or increased wake after sleep onset - have been associated with measurable decrements in high-intensity performance and decision-making capacity [7].

Importantly, sleep regulation is closely governed by circadian and thermoregulatory mechanisms that are modulated by sex hormones. Estrogen appears to exert a protective influence by enhancing REM stability and facilitating thermoregulation, whereas progesterone elevates core body temperature and contributes to sleep fragmentation [8]. Consequently, low-hormone phases - particularly the late luteal and early follicular phases - are characterized by reduced sleep efficiency, increased nocturnal awakenings, and poorer subjective sleep quality [8,9]. Emerging evidence further indicates that these hormonal fluctuations significantly alter sleep architecture, with disturbances most pronounced during low-hormone phases [7,9].

Collectively, these findings suggest that menstrual cycle physiology and sleep dynamics act as interdependent determinants of athletic performance rather than isolated variables. However, a major limitation in current literature is the lack of integrated, phase-specific analyses examining the interaction between hormonal status, sleep architecture, and performance outcomes. This gap is particularly relevant in rowing, a physiologically demanding sport in which even subtle perturbations in recovery status can materially influence high-intensity time-trial performance.

To date, no studies have comprehensively integrated menstrual cycle physiology with objective sleep metrics to explain performance variability in elite female rowers. Addressing this gap is essential to advance individualized, physiology-informed training and recovery strategies in female athletes. The present study aimed to evaluate menstrual phase-specific variations in sleep efficiency and to determine their association with 500-m rowing performance in elite female rowers.

## Materials and methods

Study design and setting

This study was designed as a prospective observational study conducted at a private sports medicine facility in South India. The study period extended from May 2025 to March 2026, providing a 10-month longitudinal monitoring window for monitoring sleep parameters and rowing performance of female athletes across phases of the menstrual cycle during nine consecutive menstrual cycles to assess the relationship between menstrual phase-induced sleep variations and performance. A repeated-measures framework was adopted to capture within-athlete variability across hormonally distinct menstrual-cycle phases.

Participants

The total number of Indian rowers undergoing training at the facility is 30, of whom 15 are male and 15 are female. Participants of this study are these 15 elite female rowers aged 18-25 years from the rowing academy affiliated with the institution. They represented a competitive, well-trained athletic cohort regularly exposed to structured rowing training and performance testing. The demographic details of participants are indicated in Table 1. Seven out of 15 participants (47%) are in the age group of 21-23 years. This is followed by five participants (33%) aged 18-20 years, and the remaining three participants (20%) are aged 24-25 years. Eligibility criteria included eumenorrheic athletes with regular menstrual cycles and active participation in organized rowing training. Athletes were excluded if they reported hormonal therapy or contraceptive use, clinically diagnosed sleep disorders, current sleep-modifying medication, or active musculoskeletal injury likely to influence sleep or performance outcomes. Written informed consent was obtained from all participants, and the study adhered to institutional ethical standards for human research.

**Table 1:** Participants' demographic details.

Demographic Details	Range	Mean ± SD
Age (years)	18-25	21.4 ± 1.9
Height (cm)	151-171	162.0 ± 6.5
Weight (kg)	57-70	62.5 ± 3.7
Training experience (years)	4-9	5.9 ± 1.4

Procedure

**Figure 1:**
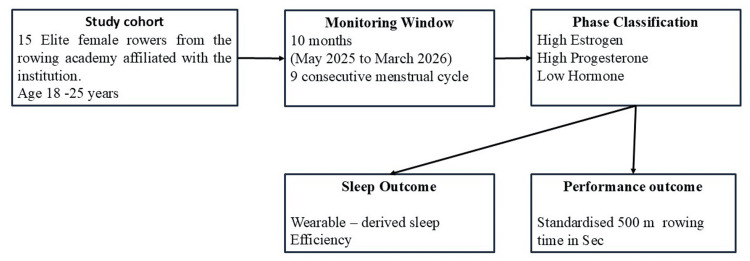
Prospective observational monitoring framework for menstrual-cycle phase classification, sleep monitoring, and 500-m performance assessment.

Participants were prospectively monitored using an integrated athlete-surveillance framework that combined menstrual-cycle tracking, wearable-derived sleep monitoring, and standardized rowing performance testing, as shown in Figure 1. Menstrual-cycle phases were classified as high-estrogen late follicular, high-progesterone early luteal, and low-hormone early follicular plus late luteal phases, based on established hormonal profiles described in the literature [3,4]. Sleep efficiency (%) was derived from Garmin (Garmin Ltd., Olathe, KS) wearable-recorded sleep metrics, including REM sleep, awake periods, light sleep, and deep sleep. Garmin devices use accelerometry and optical heart-rate-based algorithms to estimate sleep duration, sleep stages, and nocturnal awakenings. The Garmin wearable device was selected for the present study because they provide continuous, non-invasive, real-world physiological monitoring within elite training environments and has demonstrated acceptable validity for estimating sleep-related parameters [10]. Rowing performance was assessed weekly using standardized 500-m time trials conducted under consistent testing conditions using ergometer and/or on-water assessments. Both sleep efficiency and the performance time were averaged for each menstrual cycle phase for analysis.

Outcome measures

The primary outcome was 500-m rowing performance time in seconds, with lower values indicating superior performance. The principal explanatory variable was wearable-derived sleep efficiency (%). Phase-specific differences in sleep efficiency and performance were evaluated to identify potential menstrual-phase vulnerability windows and quantify the relationship between sleep quality and high-intensity rowing output.

Statistical analysis

A post-hoc power analysis was conducted based on the observed difference between high-estrogen and low-hormone phases to check the adequacy of the sample size. The observed effect size and variability indicated that the study achieved adequate statistical power (>80%) to detect phase-specific differences at a significance level of α = 0.05.

Descriptive statistics are presented as mean ± standard deviation with corresponding 95% confidence intervals (CIs). Phase-wise differences were assessed using Friedman repeated-measures analysis, with chi-square (χ²) reported as the test statistic and Kendall’s W as the effect size. Pairwise within-athlete comparisons between high-estrogen and low-hormone phases were performed using the Wilcoxon signed-rank test. Pearson correlation assessed the association between sleep efficiency and 500-m performance time using pooled phase-level observations. Statistical significance was interpreted at p < 0.05.

## Results

Phase-specific sleep efficiency and 500-m performance outcomes are summarized in Table 2. Sleep efficiency was highest during the high-estrogen or late follicular phase (96.90 ± 1.90%), declined during the high-progesterone or early luteal phase (94.20 ± 2.30%), and was lowest during the low-hormone phase (88.70 ± 2.80%). Rowing performance followed a parallel pattern. Mean 500-m time increased from 111.20 ± 2.80 s in the high-estrogen phase to 115.80 ± 2.40 s in the high-progesterone phase and 119.80 ± 3.00 s in the low-hormone phase. 

**Table 2:** Phase-specific sleep efficiency and 500-m performance outcomes. Values are presented as mean ± standard deviation (SD) with corresponding 95% confidence intervals (CI). Phase-wise differences were assessed using Friedman repeated-measures analysis, with chi-square (χ²) reported as the test statistic and Kendall’s W as the effect size. Post-hoc pairwise comparisons between high-estrogen and low-hormone phases were performed using the Wilcoxon signed-rank test.

Outcome	High-Estrogen Phase	High-Progesterone Phase	Low-Hormone Phase	Friedman χ²	p-value	Wilcoxon p-value	Effect Size Kendall’s W
Sleep efficiency (%)	96.90 ± 1.90; 95% CI: 95.85-97.95	94.20 ± 2.30; 95% CI: 92.93-95.47	88.70 ± 2.80; 95% CI: 87.15-90.25	χ² = 26.4	<0.001	0.008	0.88
500-m time (s)	111.20 ± 2.80; 95% CI: 109.65-112.75	115.80 ± 2.40; 95% CI: 114.47-117.13	119.80 ± 3.00; 95% CI: 118.14-121.46	χ² = 25.1	<0.001	0.008	0.84

Friedman repeated-measures analysis demonstrated a significant phase effect for sleep efficiency (χ² = 26.4, Kendall’s W = 0.88, p < 0.001) and 500-m performance (χ² = 25.1, Kendall’s W = 0.84, p < 0.001), indicating substantial differences across menstrual-cycle phases. The pairwise comparison between the high-estrogen and low-hormone phases using the Wilcoxon signed-rank test also showed a significant reduction in sleep efficiency and performance from the high-estrogen to low-hormone phase (Wilcoxon p = 0.008).

**Figure 2:**
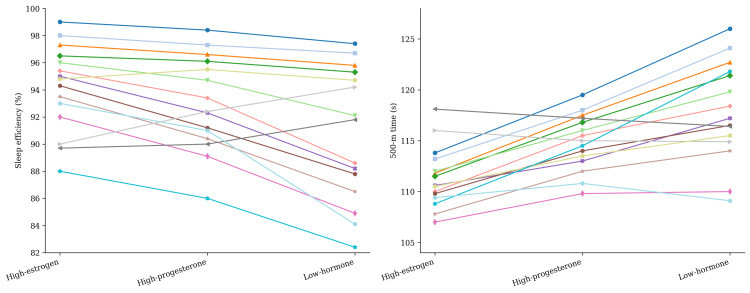
The variation of the sleep efficiency and 500-m rowing performance time of individual rowers among the three phases of the menstrual cycle.

The variation of the sleep efficiency and 500-m rowing performance time of individual rowers among the three phases of the menstrual cycle is illustrated in Figure 2. Although some of the rowers show minimal or no variation in sleep efficiency, Figure 2 clearly shows that the majority of athletes demonstrated a reduction in sleep efficiency and a corresponding increase in 500-m performance time during the low-hormone phase. Specifically, 12 out of 15 athletes (80%) demonstrated lower sleep efficiency and slower performance in the low-hormone phase compared to the high-estrogen phase.

**Figure 3:**
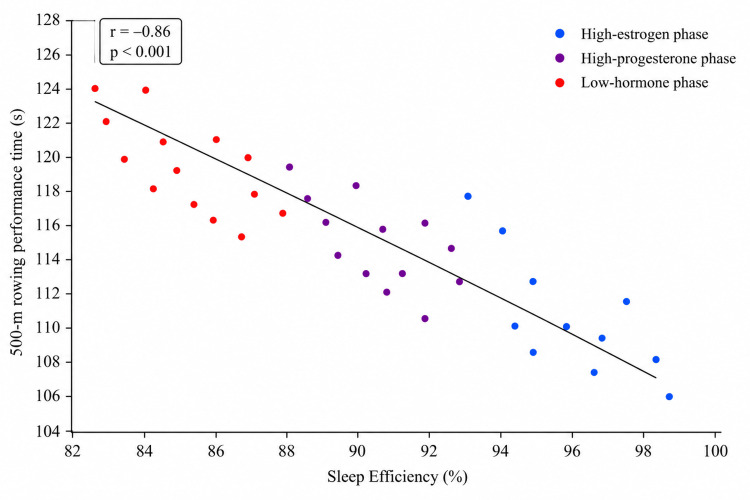
Association between sleep efficiency and 500-m rowing performance time across menstrual-cycle phases.

The regression line shown in Figure 3 clearly reveals a consistent increase in 500-m performance time with declining sleep efficiency across all menstrual-cycle phases. The high inverse correlation (r = -0.86, p < 0.001) demonstrates that slower rowing performance is closely associated with lower sleep efficiency.

## Discussion

The present study demonstrates a clear and physiologically coherent interaction between menstrual cycle phase, sleep efficiency, and rowing performance in elite female athletes. The principal finding is that phase-dependent reductions in sleep efficiency are paralleled by measurable decrements in high-intensity rowing performance, with the most adverse profile observed during low-hormone phases.

These findings are consistent with current evidence indicating that menstrual-cycle phase may influence exercise performance, although the literature remains heterogeneous and sport-specific. The observed performance decrement during low-hormone phases aligns with the concept that hormonal withdrawal may adversely influence thermoregulation, sleep continuity, perceived recovery, and physiological readiness [3,4,7,8].

A central contribution of this study is the identification of sleep efficiency as a significant predictor of rowing performance variability. This finding is supported by established evidence demonstrating that sleep quality is a critical determinant of athletic performance, with even minor perturbations in sleep architecture associated with impaired reaction time, neuromuscular function, and decision-making [7,11]. The strong inverse association observed between sleep efficiency and 500-m performance time underscores the functional importance of sleep as a modifiable performance determinant in elite athletes.

The interrelationship between menstrual cycle phase and sleep efficiency further reinforces the concept that recovery physiology in female athletes is hormonally modulated. Estrogen has been shown to exert a stabilizing influence on sleep architecture, whereas progesterone and hormonal withdrawal are associated with sleep fragmentation and reduced sleep quality [8,9]. The present findings extend this understanding by demonstrating that these sleep-related alterations have direct performance implications in a sport characterized by high physiological demand and sensitivity to recovery status.

The translational relevance of the present study lies in its integrated athlete-monitoring framework. Rather than conceptualizing menstrual cycle physiology and sleep as independent constructs, this study identifies sleep efficiency as a modifiable biomarker of recovery that may elucidate phase-related performance variability. From an applied perspective, the integration of menstrual cycle tracking with wearable-derived sleep metrics may facilitate the identification of low-hormone vulnerability windows and enable the implementation of individualized, phase-specific training, recovery, and competition strategies in elite female athletes.

The principal limitation is that hormonal phases were classified using menstrual-cycle tracking rather than biochemical confirmation, and future studies should incorporate larger cohorts, serum or urinary hormonal verification, and continuous performance monitoring across multiple training blocks. Nevertheless, the consistency of within-athlete patterns and the strength of the sleep-performance association support the relevance of this approach for female rowers.

## Conclusions

Menstrual cycle phase-specific variations significantly influence sleep efficiency and rowing performance, with low-hormone phases demonstrating the most unfavorable profile. Sleep efficiency emerged as a key physiological mediator of performance variability. By integrating menstrual cycle tracking with wearable sleep monitoring, this study addresses a critical gap in female athlete management. This approach facilitates the identification of phase-specific vulnerability windows and underpins individualized training, recovery, and competition planning in elite female athletes.
